# Oil Spill Recovery of Petroleum-Derived Fuels Using a Bio-Based Flexible Polyurethane Foam

**DOI:** 10.3390/polym17141959

**Published:** 2025-07-17

**Authors:** Fabrizio Olivito, Zul Ilham, Wan Abd Al Qadr Imad Wan-Mohtar, Goldie Oza, Antonio Procopio, Monica Nardi

**Affiliations:** 1 Department of Environmental Engineering, University of Calabria, Via P. Bucci, 87036 Arcavacata di Rende, Italy; 2 Environmental Science and Management Program, Institute of Biological Sciences, Faculty of Science, Universiti Malaya, Kuala Lumpur 50603, Malaysia; 3 Centre for Science and Environment Studies, Institute of Islamic Understanding Malaysia, 2 Langgak Tunku Off Jalan Tuanku Abdul Halim, Kuala Lumpur 50480, Malaysia; 4 Functional Omics and Bioprocess Development Laboratory, Institute of Biological Sciences, Faculty of Science, Universiti Malaya, Kuala Lumpur 50603, Malaysia; qadyr@um.edu.my; 5 Centro de Investigation y Desarollo Tecnologico en Electroquimica Parque Tecnológico Querétaro, Queretaro CP 76703, Mexico; goza@cideteq.mx; 6 Department of Health Sciences, University Magna Graecia of Catanzaro, Viale Europa—Campus Universitario S. Venuta—Loc. Germaneto, 88100 Catanzaro, Italy; procopio@unicz.it (A.P.); monica.nardi@unicz.it (M.N.)

**Keywords:** bio-based, flexible, polyurethane, oil spill, fuels

## Abstract

In this study, we tested a flexible polyurethane (PU) foam, synthesized from bio-based components, for the removal of petroleum-derived fuels from water samples. The PU was synthesized via the prepolymer method through the reaction of PEG 400 with L-lysine ethyl ester diisocyanate (L-LDI), followed by chain extension with 2,5-bis(hydroxymethyl)furan (BHMF), a renewable platform molecule derived from carbohydrates. Freshwater and seawater samples were artificially contaminated with commercial diesel, gasoline, and kerosene. Batch adsorption experiments revealed that the total sorption capacity (S, g/g) of the PU was slightly higher for diesel in both water types, with values of 67 g/g in freshwater and 70 g/g in seawater. Sorption kinetic analysis indicated that the process follows a pseudo-second-order kinetic model, suggesting strong chemical interactions. Equilibrium data were fitted using Langmuir and Freundlich isotherm models, with the best fit achieved by the Langmuir model, supporting a monolayer adsorption mechanism on homogeneous surfaces. The PU foam can be regenerated up to 50 times by centrifugation, maintaining excellent performance. This study demonstrates a promising application of this sustainable and bio-based polyurethane foam for environmental remediation.

## 1. Introduction

In the era of rapid electrification of road vehicles, conventional liquid fuels continue to occupy a significant share of the global energy market, especially in sectors where electrification faces technological or economic challenges [1]. Kerosene remains the primary aviation fuel, widely used in jet engines due to its high energy density and suitable combustion properties [2]. For road transportation, gasoline is predominantly used in hybrid vehicles, providing flexibility between internal combustion and electric drive modes [3]. In the maritime sector, the industry is undergoing a critical transition from the traditional use of heavy fuel oil (HFO) toward cleaner alternatives such as marine diesel oil (MDO) and low-sulfur fuels, driven by increasingly stringent environmental regulations like the IMO 2020 sulfur cap [4]. These measures aim to reduce emissions of sulfur oxides (SO_x_), nitrogen oxides (NO_x_), and particulate matter, mitigating the environmental footprint of shipping [5]. Emerging alternative fuels, including liquefied natural gas (LNG) and biofuels, are also gaining attention as part of decarbonization strategies in the maritime industry [6]. Every year, tons of fuels are transported across seas and oceans, and accidental spills frequently cause catastrophic damage with irreversible effects on marine ecosystems [7]. To mitigate these impacts, various oil removal techniques from aqueous environments have been developed, broadly categorized into chemical, physical, and biological methods [8]. Chemical methods include sorption, degradation, coagulation, flocculation, and the use of surfactants [9,10,11]. Among these, chemical sorption remains one of the most widely employed techniques due to several advantages: the materials used are often environmentally benign, can be regenerated for multiple uses, exhibit strong affinity for oil phases, and demonstrate high removal efficiency [12,13]. Advances in bio-based absorbents have further enhanced the sustainability and efficacy of these approaches, aligning with increasing environmental regulations and the need for eco-friendly remediation [14,15]. The global production of polyurethane (PU) in 2023 is estimated to be around 7 to 8 million metric tons, representing a significant but smaller portion of the overall worldwide plastic production, which reached approximately 413.8 million metric tons in 2023 [16]. Polyurethanes are synthesized primarily through the reaction of diisocyanates with polyols and find wide application in foams, coatings, adhesives, and elastomers [17]. PU foams constitute the largest segment of the market, with rigid and flexible foams accounting for nearly 65–70% of total polyurethane production due to their thermal insulation and cushioning properties [18]. The remaining PU production is distributed among coatings, adhesives, and elastomers, utilized in automotive, construction, furniture, and electronic industries [19]. Despite the extensive use, environmental concerns linked to PU production and disposal have prompted research into bio-based polyols, reagents, and enhanced recycling technologies. For instance, recent advances demonstrate the potential for synthesizing polyols from renewable resources like lignin and vegetable oils, reducing reliance on fossil-derived chemicals [20,21]. Moreover, chemical recycling approaches aiming at depolymerization are increasingly explored to recover raw materials and minimize PU waste [22]. Given the global push toward sustainability, these developments are expected to impact the PU market significantly, balancing demand growth with environmental responsibility.

This study explores the potential of a bio-based flexible-PU foam as an efficient and reusable sorbent for the removal of petroleum-derived pollutants from aquatic environments. By investigating the material’s performance in conditions simulating real-world oil spills, the work aims to contribute to the development of environmentally responsible solutions for oil spill remediation.

This work is innovative in several aspects:

- It uses a bio-based polyurethane derived from renewable sources, offering a sustainable alternative to traditional fossil-derived foams.
- It demonstrates high sorption capacity (up to 70 g/g) for three common fuels (diesel, gasoline, and kerosene), in both freshwater and seawater, which is uncommon in the literature.
- The foam exhibits excellent reusability, maintaining sorption performance for up to 50 regeneration cycles using a simple and non-destructive method (centrifugation).
- It introduces a material with practical relevance for oil spill remediation, supporting circular economy goals.

## 2. Materials and Methods

### 2.1. Materials

All reagents used in this study were of analytical or high-purity grade. Tetrahydrofuran (THF) was obtained from Carlo Erba (Milan, Italy) and was freshly distilled prior to use. 2-Pentanone (MPK) was purchased from Sigma Aldrich (St. Louis, MO, USA) and used as received. D-(−)-Fructose (99% purity) and ytterbium triflate (99.9%) were sourced from Thermo Fisher Scientific (Waltham, MA, USA). Choline chloride (99%) was acquired from Sigma Aldrich (St. Louis, MO, USA). Sodium borohydride (95%) and iodine (analytical grade) were also provided by Sigma Aldrich (St. Louis, MO, USA). Polyethylene glycol (PEG 400), with 99% purity, was obtained from Thermo Fisher Scientific (Waltham, MA, USA). L-Lysine ethyl ester diisocyanate (L-LDI, 97%) was supplied by Sigma Aldrich (St. Louis, MO, USA). Sodium chloride and anhydrous sodium sulfate were both purchased from Thermo Fisher Scientific (Waltham, MA, USA) and used without further purification.

### 2.2. Methods

#### 2.2.1. Synthesis of HMF from D-Fructose

A modified version of a previously published method was used for the synthesis of 5-hydroxymethylfurfural (HMF) starting from D-fructose [23]. In a 100 mL single-neck round-bottom flask, 5 g of fructose was combined with choline chloride (1.5 equivalents) and ytterbium triflate (4 mol% relative to fructose). Then, 60 mL of 2-pentanone was added as the solvent. The reaction mixture was equipped with a condenser and heated to 130 °C under reflux conditions at atmospheric pressure, with constant stirring for 2 h. Upon completion, the mixture was decanted, dried using anhydrous sodium sulfate, and filtered through a sintered glass funnel. The solvent was evaporated under reduced pressure to obtain a yellowish oil. The product, isolated in 88% yield, was characterized by GC-MS and HPLC, showing no detectable impurities or side products (see the Supporting Information File). A single peak appears in the mass chromatogram, with a mass spectrum with relative *m*/*z* 126, this is the molecular ion peak [M]^+^, representing the intact HMF molecule. Other typical fragment ions are *m*/*z* 97 and 109, formed by the loss of functional groups like water or the hydroxymethyl group. In the HPLC chromatogram, however, the single peak appears at the same retention time as the HMF standard (Figure S5).

#### 2.2.2. Synthesis of BHMF from HMF

A total of 3 g of HMF, previously synthesized as described, was dissolved in 60 mL of dry tetrahydrofuran (THF) in a 100 mL single-neck round-bottom flask. Sodium borohydride (NaBH_4_) was added gradually in an amount corresponding to 25% by weight relative to the expected BHMF product. The reaction was carried out under open-flask conditions to allow for the release of hydrogen gas generated during the reduction. After stirring for one hour, iodine was slowly introduced (30% by weight relative to BHMF), resulting in further hydrogen evolution. Subsequently, any unreacted sodium borohydride was neutralized with 1N aqueous HCl. The mixture was then filtered through a sintered glass funnel, dried over anhydrous sodium sulfate, and the solvent removed under reduced pressure. The resulting polyol was obtained as a colorless oil with an 85% yield [14]. The product was confirmed to be BHMF as the major component via HPLC and GC-MS analyses. (See Supporting Information File). In the mass chromatogram, a single peak appears, whose relative mass spectrum gives a molecular ion peak [M]^+^ of *m*/*z* 128, and the relative fragments as: *m*/*z* 110 (loss of H_2_O), *m*/*z* 97 or *m*/*z* 99, depending on fragmentation of the hydroxymethyl groups or ring cleavage (Figure S6). In the HPLC chromatogram, however, the single peak appears at the same retention time as the BHMF standard (Figure S6).

#### 2.2.3. Prepolymer Synthesis

Water was utilized as a blowing agent at a concentration of 5.6 wt% relative to PEG 400. PEG 400, distilled water, and NaCl catalyst (4 wt% based on the initial PEG 400 amount) were combined in a plastic container and stirred using a mechanical mixer. L-Lysine ethyl ester diisocyanate (L-LDI) was then introduced at a molar ratio of 2.5:1 with respect to PEG 400, and the mixture was thoroughly agitated. The blend was heated to 70 °C and maintained for one hour until the diisocyanate was fully consumed. The resulting prepolymer appeared as a colorless gel [14]. The reaction progress was monitored by FT-IR spectroscopy by tracking the isocyanate peak corresponding to free isocyanate groups in the prepolymer. The isocyanate content was monitored with a titration test using bromophenol blue indicator and hydrochloric acid, until a constant value was reached (see Supporting Information).

#### 2.2.4. Polyurethane Synthesis Using BHMF for Chain Extension

**Figure 1:**
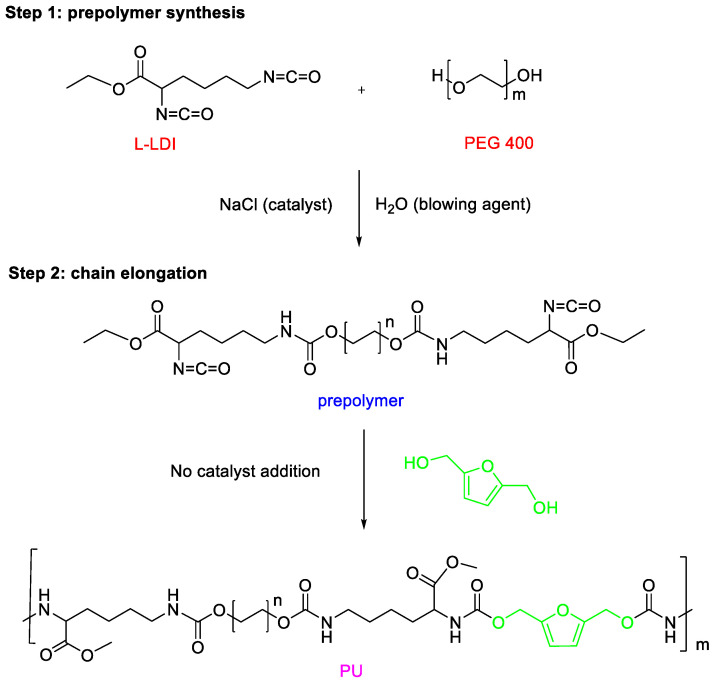
Synthesis of the bio-based flexible-PU foam [14].

BHMF was incorporated at 30 wt% relative to the prepolymer, and the mixture was vigorously stirred mechanically for several minutes in the same plastic container. Subsequently, the mixture was transferred into a steel mold, which was sealed under pressure at ambient temperature. From the superimposed FT-IR spectrum, the disappearance of the isocyanate signal is evident in the region (2250–2270 cm^−1^), confirming the completeness of the polymerization reaction (Figure S7). After 24 h, the resulting polyurethane foam was formed as a flexible white sponge and was left to air dry overnight. The synthesis of the bio-based flexible-PU foam was carried out following the procedure described in our previous work [14]. The reaction scheme is illustrated in Figure 1.

#### 2.2.5. Adsorption Capacity Test in Oil/Freshwater System

The adsorption capacity within the oil/freshwater system was evaluated following the method described elsewhere [12]. Cubic samples of the sorbent were immersed in the oil/water mixture under stirring, using various concentrations established in this study. After the specified contact time, the sorbent was removed and placed in a centrifuge tube (GT1R Centrifuge, Fisher Scientific) to separate the absorbed oil. The samples were centrifuged at 500 rpm for 1 min, allowing the oil to settle at the bottom. This regeneration process can be repeated up to 50 cycles. (All the single regeneration tests are reported in Figure S1 of the Supporting Information File). The resulting biphasic mixture, composed mainly of fuel with a small water content, underwent liquid-liquid extraction using three portions of petroleum ether to isolate the organic phase. The combined organic extracts were dried over anhydrous sodium sulfate, filtered through a sintered glass funnel into a one-neck round-bottom flask, and the solvent was removed under reduced pressure. The oil adsorption capacity was then calculated using Equation (1):

(1) $$S(g/g)=\frac{M_{t}-M_{0}}{S_{0}}$$

where *M*_0_ represents the weight of the reaction flask alone, *M_t_* denotes the combined weight of the flask and the oil collected after the process, and *S*_0_ is the initial weight of the sorbent.

#### 2.2.6. Adsorption Capacity Test in Oil/Seawater System

The adsorption capacity was determined using the same procedure described for the oil/freshwater system. The influence of varying ionic strength on sorption performance was investigated. Different solutions were prepared by adding oil to the surface of a synthetic seawater substitute, formulated according to the ASTM D1141-98 standard [24].

#### 2.2.7. Characterization

##### FT-IR

FT-IR spectra were recorded using a Nicolet Impact 410 FT-IR Spectrometer (SpectraLab Scientific Inc., 38 McPherson St., Markham, ON, Canada L3R 3V6). Details are provided in the Supporting Information.

##### GC-MS

Organic solvent-soluble products were analyzed with GC-MS using a Thermo Scientific TSQ 7000 system (Waltham, MA, USA), equipped with a ROTI^®^Cap-1701 MS capillary column (30 m length, 0.25 mm diameter, 0.25 µm film thickness). The instrument operated in split mode with helium as the carrier gas at a flow rate of 1 mL/min, coupled with a quadrupole mass detector. Further information is available in the Supporting Information.

##### HPLC

Products soluble in the aqueous phase were analyzed on a Thermo Fisher UltiMate 3000 HPLC system (Waltham, MA, USA) featuring an isocratic pump and UV-vis detector. The column used was a C18 ODS-A reversed-phase column (120 Å, 150 × 3 mm, 3 µm particle size; Thomas Scientific, Chadds Ford, PA, USA). The reference wavelength was set to 360 nm with a peak width of 100, while the analysis wavelength was 385 nm with a peak width of 8 mm. The mobile phase consisted of methanol (49.99%) and acetonitrile (50%) under isocratic conditions at a flow rate of 1 mL/min. The sample injection volume was 20 μL. Refer to the Supporting Information for more details.

##### SEM

Scanning electron microscopy images were acquired using a Hitachi SU3800 (Hitachi High-Tech, Tokyo, Japan). Additional information can be found in the Supporting Information, Figure S8, in which the SEM image demonstrates a uniform morphology of a classical porous material.

## 3. Results and Discussion

This bio-based PU foam was evaluated for its ability to adsorb common petroleum-derived fuels. Initially, we examined both the total sorption capacity and the reusability of the foam. Additionally, the sorption mechanism was studied by analyzing the oil uptake kinetics in diesel/water, gasoline/water, and kerosene/water systems until equilibrium was achieved. Pseudo-first-order and pseudo-second-order kinetic models were applied, as they are widely used for this type of material [12,25]. Subsequently, the sorption capacity was assessed at varying oil concentrations, and the experimental results were compared to conventional adsorption isotherm models, specifically Langmuir and Freundlich. All experiments were carried out using both artificially contaminated freshwater and seawater samples.

### 3.1. Total Sorption Capacity of PU in Diesel/Water, Gasoline/Water, and Kerosene/Water Systems in Batch

**Figure 2:**
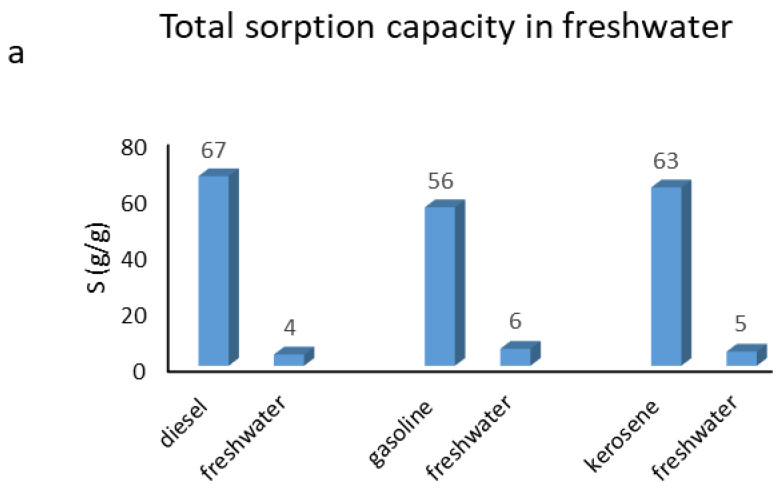

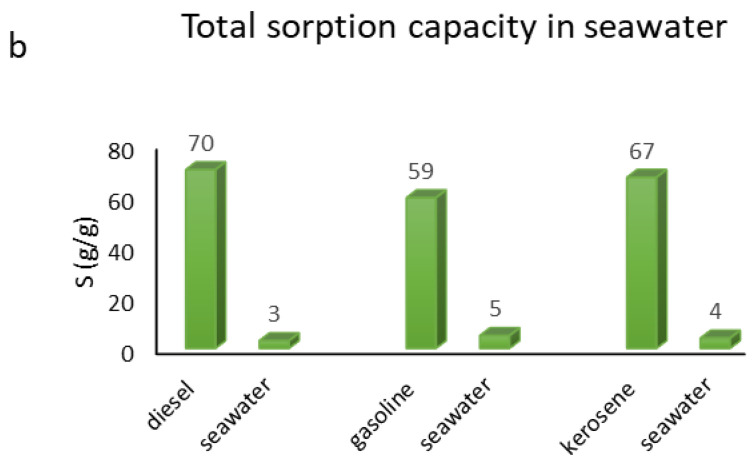
Total sorption capacity of PU in freshwater systems (**a**) and total sorption capacity of PU in seawater systems (**b**), starting from a solution of 80 g/L.

We evaluated the total sorption capacity S (g/g) of the PU foam using an 80 g/L solution of oil-contaminated water. Following the initial test, we observed that sorption equilibrium was reached within 60 s. This rapid uptake suggests that simultaneous sorption is more likely than competitive sorption. The PU foam can be regenerated up to 50 times by centrifugation after each batch test. Remarkably, the sorption capacity remained stable throughout the regeneration cycles, with no evidence of material deformation or flaking. Figure 2 presents the plots of S (g/g), showing the quantities of sorbed oil and water. The separation of oil and water was achieved using the method described in Section 2.2.6.

The data presented in Figure 2 confirm the high hydrophobicity of the PU foam. In fuel-contaminated freshwater samples, the amount of water sorbed ranged from only 4% to 6%. The sorption capacities for diesel were 67 g/g in freshwater and 70 g/g in seawater; for gasoline, 56 g/g in freshwater and 59 g/g in seawater; and for kerosene, 63 g/g in freshwater and 67 g/g in seawater.

The PU foam consistently showed higher affinity for diesel across all tests, likely due to diesel’s greater density compared to gasoline and kerosene. This higher density may enhance retention within the polymer pores, in addition to the contribution of hydrophobic interactions [12].

These results suggest that both physical and chemical properties of the oils influence the sorption performance of the PU foam. The higher sorption observed in seawater compared to freshwater for all tested fuels may be attributed to the increased ionic strength and presence of salts, which can enhance phase separation and promote oil sorption by reducing the oil–water interfacial tension.

Moreover, the low percentage of water uptake reinforces the material’s selectivity toward hydrophobic compounds, making it particularly suitable for oil spill remediation in marine and freshwater environments. The consistent sorption capacity over multiple regeneration cycles further supports its potential for sustainable and cost-effective application in real-world scenarios.

To gain further insights into the sorption mechanism, we fitted the experimental data to both pseudo-first-order and pseudo-second-order kinetic models and intra-particle diffusions (Weber–Morris) models, as well as to Langmuir and isotherms, which are discussed in detail in the following section.

### 3.2. Sorption Kinetics of PU in Freshwater and Seawater Systems in Batch

Assuming that the pseudo-first-order, pseudo-second-order, and intra-particle diffusion models are appropriate for this type of study, we evaluated the sorption kinetics using the corresponding equations, reported below:

Pseudo-first-order model:

(2) $$\ln\;(\frac{q_{e}}{q_{e}-q_{t}})=k_{1}t$$

Pseudo-second-order model:

(3) $$\frac{t}{q_{t}}=\frac{t}{q_{e}}+\frac{1}{k_{2}q_{e}^{2}}$$

Intra-particle diffusion model:

(4) $$q_{t}=k_{id}\cdot t^{1/2}+C$$

where q_e_ is the amount of oil sorbed at equilibrium (g/g); q_t_ is the amount of oil sorbed at time *t* (g/g); and *k*_1_, *k*_2_, and *k*_id_ are the rate constants for the first-, second-order, and intra-particle diffusion models, respectively. C is the intercept related to the boundary layer effect for the intra-particle diffusion model (i.e., external mass transfer resistance).

In all experiments, equilibrium was reached within 60 s. Table 1 presents both the experimental and theoretical q_e_ values (g/g), as well as the corresponding kinetic parameters for the PU foam in the sorption of fuel oils from freshwater and seawater samples. The experimental q_e_ values were obtained from batch sorption tests.

We employed the pseudo-first-order, pseudo-second-order, and intra-particle diffusion kinetic models to interpret the sorption mechanism. The pseudo-first-order model describes processes characterized by linear sorption equilibrium, time-dependent kinetics that are not influenced by solute concentration, and systems that rapidly reach equilibrium [12,26]. Such behavior typically occurs during the initial stages of the sorption process.

The pseudo-second-order model is more suitable for systems where chemical interactions dominate the sorption mechanism, as opposed to purely physical processes such as inclusion, occlusion, or diffusion [26,27]. This model assumes that the rate-limiting step may involve valence forces through the sharing or exchange of electrons between the sorbent and sorbate.

The intra-particle diffusion model, on the other hand, was found not to be the rate-limiting step in this case. This conclusion is supported by the non-linear trend observed in the corresponding plots, the fact that the regression lines do not pass through the origin, and the intercept values C ≠ 0, as shown in the Supporting Information File.

**Figure 3:**
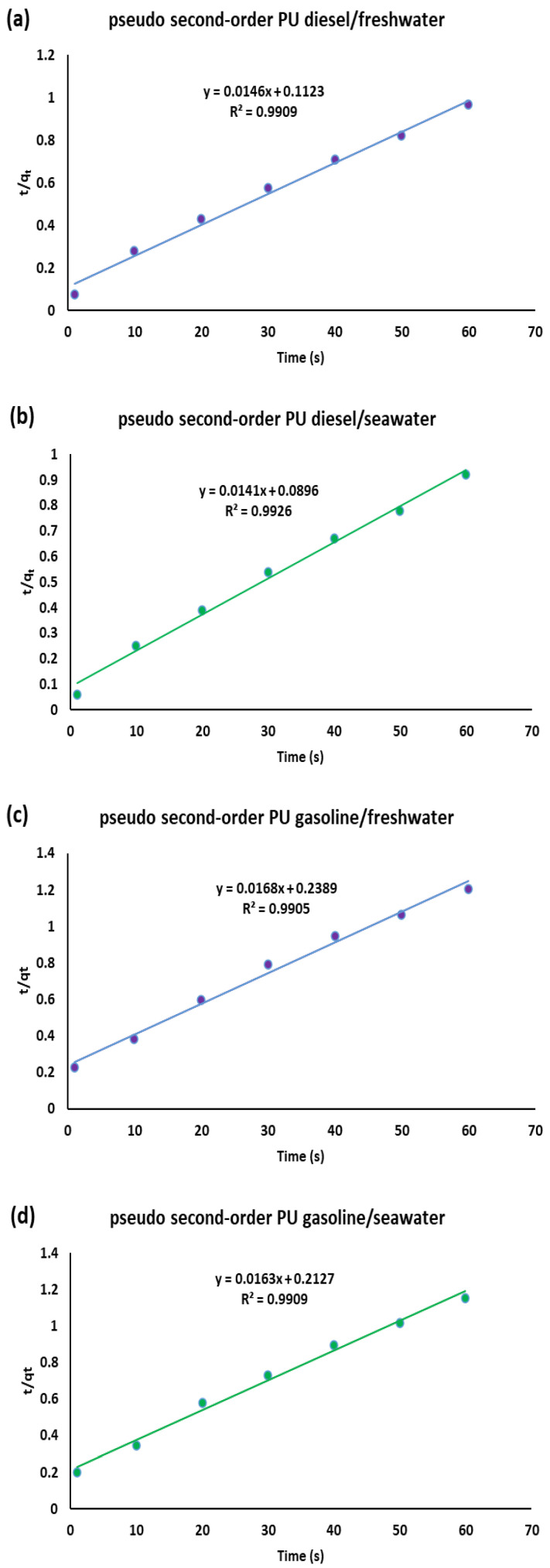

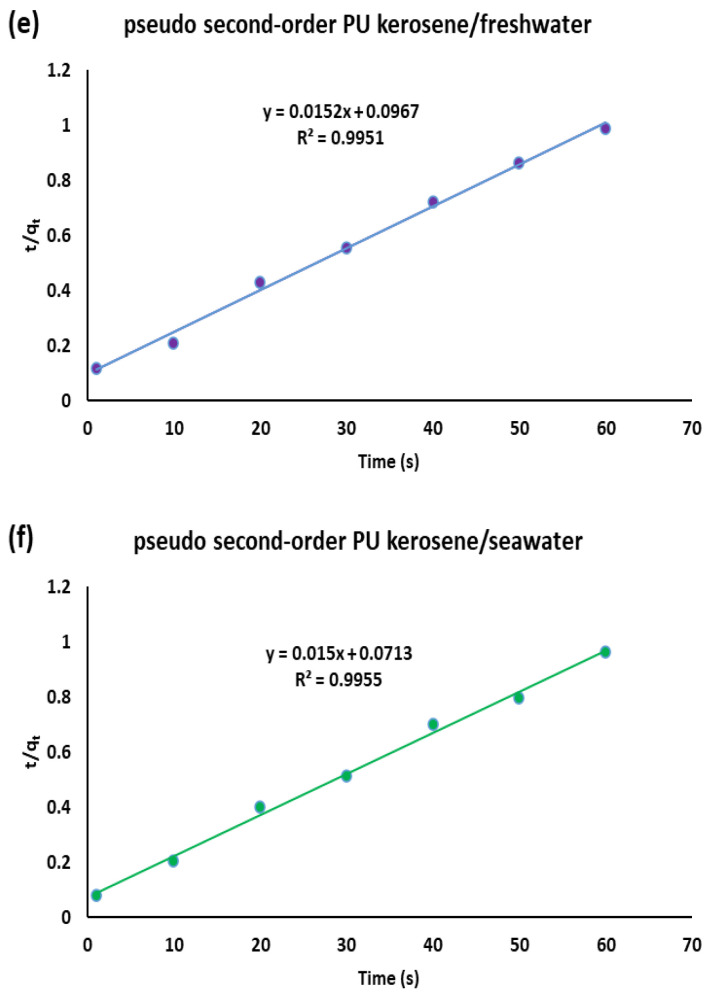
Pseudo second-order sorption linear fitting of PU in diesel/freshwater (**a**) and diesel/seawater (**b**) systems. Pseudo second-order sorption linear fitting of PU in gasoline/freshwater (**c**) and gasoline/seawater (**d**) systems. Pseudo second-order fitting of PU in kerosene/freshwater (**e**) and kerosene/seawater (**f**) systems.

Figure 3 presents the kinetic plots corresponding to the pseudo-second-order model for the sorption of gasoline, diesel, and kerosene onto polyurethane (PU) in both freshwater and seawater samples.

We employed the pseudo-first-order, pseudo-second-order, and intra-particle diffusion kinetic models to investigate the sorption mechanism of the polyurethane (PU) foam. These models help distinguish between different rate-limiting processes, namely, physisorption, chemisorption, and diffusion-limited uptake, and thus provide insights into how the structural and chemical features of the material influence its performance.

The pseudo-first-order model typically describes systems where sorption occurs rapidly and is predominantly physical. While our data show moderate correlation with this model (R^2^ values between 0.91 and 0.96), the poor agreement between experimental and calculated q_e_ values suggests it does not fully capture the dominant mechanism.

The pseudo-second-order model, by contrast, showed excellent correlation (R^2^ > 0.99) and accurate prediction of the q_e_ values across all fuel types and aqueous environments, strongly indicating that chemisorption is the rate-limiting step in the system. This suggests that the interaction between the PU foam and the hydrocarbons likely involves chemical bonding or strong intermolecular forces, possibly through polar interactions or hydrogen bonding between the BHMF-derived furan rings and fuel components. 

The intra-particle diffusion model plots were non-linear and did not pass through the origin, as also confirmed by non-zero intercept values (C ≠ 0). This indicates that intra-particle diffusion is involved but not rate-limiting, and that external boundary layer effects also play a significant role in the early stages of sorption.

Taken together, these kinetic results highlight several key structure–property relationships:

- The dominance of pseudo-second-order behavior suggests that modifying the chemical composition of the PU, especially its polar and aromatic groups, could directly tune sorption kinetics and selectivity. For instance, increasing the content of BHMF may enhance chemisorption-driven uptake, particularly for high-viscosity pollutants like diesel.
- The secondary role of intra-particle diffusion implies that porosity and surface area are less limiting than surface chemistry, at least within the short contact times tested. This is supported by the fact that equilibrium was consistently reached within 60 s, a performance metric that is important for real-world spill applications.
- The higher sorption capacities observed in seawater across all fuels suggest that ionic strength plays a synergistic role, possibly enhancing phase separation at the water–oil interface. This insight could be useful in designing foams for marine applications, where salinity is an intrinsic factor.

Overall, the kinetic modeling not only confirms the efficiency of the PU foam but also provides strategic guidance for future material design: focusing on functional group optimization and tuning the chemical environment at the polymer surface could lead to even faster and more selective sorption behavior. This level of understanding is critical when developing advanced sorbents for specific pollutants or environmental conditions.

### 3.3. Adsorption Isotherms in Freshwater and Seawater Systems in Batch

To gain a deeper understanding of the sorption behavior, we evaluated which isotherm model, between Langmuir and Freundlich, best represents the experimental data obtained for the gasoline, diesel, and kerosene systems in freshwater and seawater. The Langmuir isotherm assumes monolayer adsorption onto a homogeneous surface with uniformly distributed functional groups, where no chemical reaction occurs between the adsorbate and the adsorbent [28,29]. The Langmuir model is described by the following equation:

(5) $$q_{e}=q_{max}\frac{K_{L}C_{e}}{1+K_{L}C_{e}}$$

in which q_max_ is the maximum sorption capacity (g/g), q_e_ is the quantity of oil sorbed at the equilibrium (g/g), C_e_ is the oil concentration at the equilibrium (g/L), and K_L_ is the Langmuir constant related to the affinity of the binding sites (L/g). To further evaluate the nature of the adsorption process, we calculated the dimensionless separation factor R_L_, a key parameter derived from the Langmuir model [27], defined as:

(6) $$R_{L}=\frac{1}{1+K_{L}C_{0}}$$

where *K_L_* (L/g) represents the Langmuir constant and *C*_0_ is the initial oil concentration (g/L). This parameter is crucial as it indicates the nature of the adsorption process: unfavorable if *R_L_* > 1, linear when *R_L_* = 1, favorable if 0 < *R_L_* < 1, or irreversible when *R_L_* = 0 [28].

The Freundlich isotherm model describes non-ideal and reversible adsorption on heterogeneous surfaces, where adsorption occurs in multilayers rather than a single monolayer. This model assumes that adsorption sites are not equivalent and that the binding strength varies across the surface. The Freundlich equation is expressed as:

(7) $$q_{e}=K_{F}C_{e}^{1/n}$$

where *K_F_* denotes the Freundlich equilibrium constant associated with the adsorption capacity, *n* indicates the affinity between the oil and the sorbent material, and *q_e_* represents the amount of oil adsorbed per unit weight of sorbent at equilibrium [28,29].

**Figure 4:**
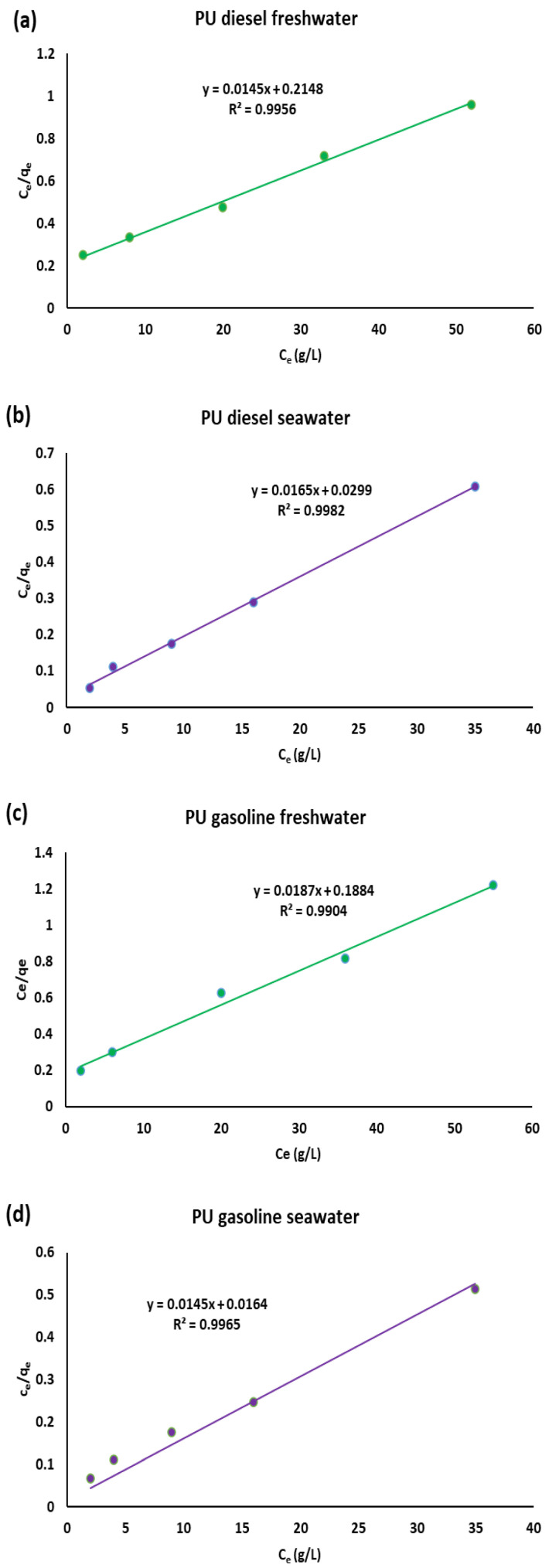

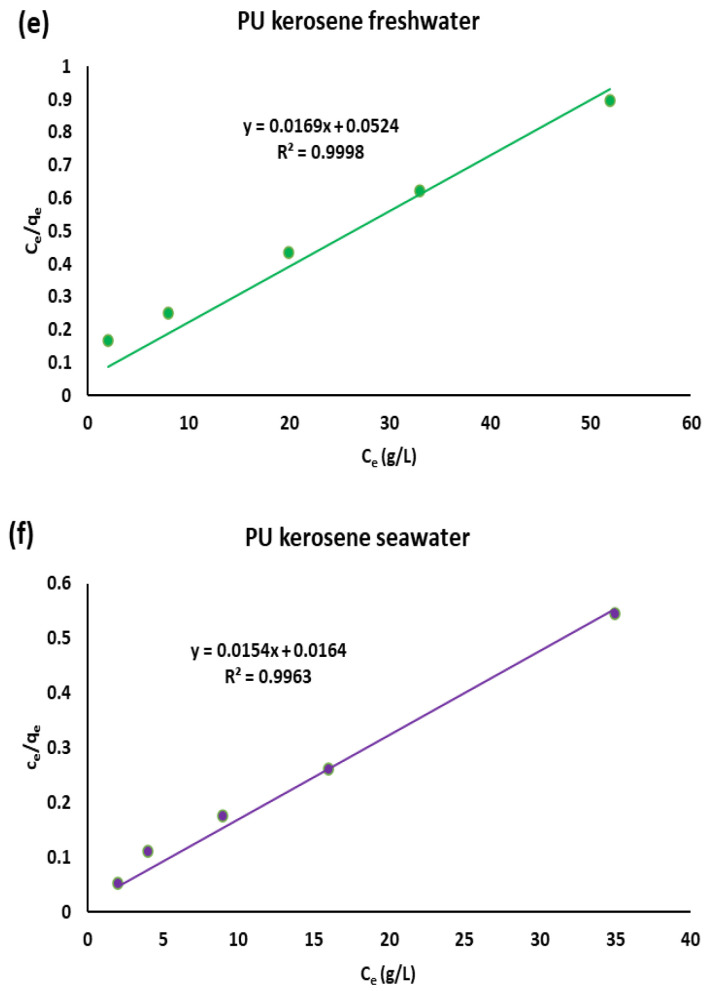
Langmuir models for the sorption of diesel, gasoline, and kerosene onto PU in freshwater (**a**,**c**,**e**) and in seawater (**b**,**d**,**f**).

We investigated the sorption behavior of polyurethane (PU) in gasoline, diesel, and kerosene within both freshwater and seawater systems, using a range of initial oil concentrations: 40, 60, 80, 100, and 120 g/L. The best-fitting curves for the Langmuir isotherm model, along with the corresponding experimental data, are presented in Figure 4.

The plots reported in Figure 4 and the relative correlation coefficients R^2^ of the following Table 2 proved that Langmuir is the best model for this type of process. The Langmuir model is relative to monolayer adsorption on a surface with a finite number of functional groups that are organized in a homogeneous manner [29]. The Freundlich model did not furnish good correlation coefficients, proving that this model is more suitable for multilayer and heterogeneous adsorption (Supporting Information File). We reported the obtained parameters of the two models in Table 2.

From Table 2, the maximum sorption capacity (*q_m_*) values derived from the Langmuir equation closely matched the experimental results obtained from batch tests. Similarly, the Langmuir constant (*K_L_*), which relates to the sorption energy or the affinity of the sorbent for the oil, also showed good agreement, indicating favorable sorption. This behavior of the Langmuir model was consistent across diesel/water, gasoline/water, and kerosene/water mixtures.

A key feature of the Langmuir isotherm is described by the dimensionless separation factor, *R_L_*. This parameter classifies the isotherm as unfavorable (*R_L_* > 1), linear (*R_L_* = 1), favorable (0 < *R_L_* < 1), or irreversible (*R_L_* = 0) [28,29].

In all cases tested, the *R_L_* values indicated favorable adsorption (see Supporting Information), confirming that the Langmuir isotherm accurately represents the sorption process.

### 3.4. A Comparison with Literature Data

To better assess the effectiveness of the synthesized bio-based flexible-PU foam, its performance was compared with similar materials reported in recent studies. Table 3 summarizes the sorption capacities of various polyurethane-based foams, including those derived from renewable resources or modified with natural additives. The comparison focuses on sorption capacity (g/g), the type of oil tested, and regeneration.

As shown in Table 3, the PU foam developed in this study demonstrates superior sorption capacity toward petroleum-derived fuels, ranging from 67 g/g to 70 g/g, depending on the fuel type and water matrix. These values are significantly higher than those reported for other bio-based PU foams, which typically exhibit sorption capacities between 8.1 g/g and 65 g/g. For example, a sunflower-oil-based PU with bagasse fibers achieved around 15.2 g/g [30]. A recent study reported a bio-based PU with a sorption capacity of 62–65 g/g for motor oil, which is among the highest in the literature but still lower than the values obtained in our work [12].

Notably, the present material also maintains excellent reusability, with up to 50 regeneration cycles achieved via simple centrifugation, without loss of performance. This contrasts with the majority of the other studies, where regeneration is often limited to 20–30 cycles or not reported at all.

The outstanding performance of our PU foam may be attributed to its tailored chemical structure, which enhances both hydrophobic interactions and surface affinity toward hydrocarbons, and to its porous morphology that facilitates rapid capillary uptake. Moreover, the use of bio-based monomers and the proven biodegradability under natural conditions make this material a competitive and sustainable alternative to fossil-derived sorbents.

These findings support the potential scalability and environmental relevance of the proposed PU foam for real-world applications in oil spill response and water remediation.

## 4. Conclusions

In this study, we evaluated the sorption capacity of a bio-based flexible polyurethane (PU) foam for petroleum-derived pollutants. The PU foam was tested with diesel, gasoline, and kerosene in both freshwater and seawater artificial contamination samples under batch conditions. The results showed excellent sorption performance, with slightly better results for diesel and in seawater compared to freshwater.

The collected oil phases were recoverable by centrifugation, and the foam demonstrated the ability to be regenerated up to 50 times without loss of sorption capacity. Sorption kinetics followed a pseudo-second-order model with high correlation coefficients, indicating that chemical interactions are the dominant sorption mechanism. Equilibrium data were best described by the Langmuir isotherm model, suggesting monolayer adsorption on a homogeneous foam surface.

This study highlights the potential of sustainable and bio-based materials to achieve sorption properties comparable to or superior to petroleum-derived analogues. The foam’s excellent performance confirms its suitability for environmental remediation and pollution control applications.

Future work will focus on scaling up production, testing performance in dynamic flow systems and real spill scenarios, and assessing sorption behavior in complex pollutant mixtures. Additionally, surface modifications or incorporation of functional additives could further enhance sorption selectivity and broaden the range of target contaminants.

## Figures and Tables

**Table 1:** Maximum oil sorption capacities at equilibrium, rate constants, and correlation coefficients evaluated at 25 °C.

PU Freshwater	Parameters	Diesel	Gasoline	Kerosene
q_e_ (g/g) experimental		67	56	63
Pseudo first-order	R^2^	0.9476	0.9607	0.9101
	q_e_ (g/g)	1.13	1.39	1.35
	k_1_	0.0686	0.0647	0.0606
Pseudo second-order	R^2^	0.9909	0.9905	0.9951
	q_e_ (g/g)	68.49	59.52	65.7
	k_2_	0.0019	0.0008	0.0024
Intra-particle diffusion	R^2^	0.8356	0.7241	0.7264
	C	13.955	15.462	14.5
	k_id_	4.9938	4.677	4.7812
**PU Seawater**	**Parameters**	**Diesel**	**Gasoline**	**Kerosene**
q_e_ (g/g) experimental		70	59	67
Pseudo first-order	R^2^	0.9547	0.9551	0.9565
	q_e_ (g/g)	1.22	1.01	1.17
	k_1_	0.06	0.0591	0.0612
Pseudo second-order	R^2^	0.9926	0.9909	0.9955
	q_e_ (g/g)	70.9	61.35	66.6
	k_2_	0.0022	0.0012	0.0031
Intra-particle diffusion	R^2^	0.8724	0.8154	0.8161
	C	14.186	14.436	14.467
	k_id_	4.9527	4.8266	4.7855

**Table 2:** Regression analysis of oil sorption by PU. Parameters estimated using Langmuir and Freundlich models.

PU Freshwater	Parameters	Diesel	Gasoline	Kerosene
Isotherm				
Langmuir model	K_L_	0.067	0.099	0.322
	q_m_ (g/g)	68.96	53.48	59.17
	R^2^	0.9956	0.9904	0.9998
Freundlich model	K_F_	641.21	953.23	722.77
	n	3.66	2.74	3.86
	R^2^	0.9197	0.9353	0.8748
**PU Seawater**	**Parameters**	**Diesel**	**Gasoline**	**Kerosene**
**Isotherm**				
Langmuir model	K_L_	0.552	0.884	0.939
	q_m_ (g/g)	60.61	68.96	64.9
	R^2^	0.9982	0.9965	0.9963
Freundlich model	K_F_	537.28	979.94	538.52
	n	3.23	2.68	3.27
	R^2^	0.9743	0.9472	0.9719

**Table 3:** Comparison of oil sorption capacities of PU-based materials reported in recent literature.

Material	Sorption Capacity (g/g)	Oil Type	Regeneration	Reference
Bio-based PU (this study)	56–70	Diesel, gasoline, kerosene	50	This study
Sunflower-oil-based PU with bagasse fibers	15.2	Diesel	Not specified	[30]
Magnetic and Hydrophobic Composite PU	32–40	Peanut Oil	6	[31]
Coconut-oil-based super-oleophilic PU	14.89–24.65	Vegetable oil, Engine oil and others	Up to 20	[32]
PU with algae-derived activated carbon (PUF1B)	53	Diesel	Not specified	[33]
Bio-based PU	49–65	Diesel and gasoline	50	[12]

## Data Availability

The original contributions presented in this study are included in the article/Supplementary Material. Further inquiries can be directed to the corresponding authors.

## Supplementary Materials

The following supporting information can be downloaded at: https://www.mdpi.com/article/10.3390/polym17141959/s1, Figure S1. Regeneration tests in fuel/freshwater and seawater systems; Figure S2. (a) Freundlich plot for diesel/freshwater; (b) Freundlich plot for diesel/seawater; (c) Freundlich plot for gasoline/freshwater; (d) Freundlich plot for gasoline/seawater; (e) Freundlich plot for kerosene/freshwater; (f) Freundlich plot for kerosene/seawater; Figure S3. FT-IR Spectrum of PEG 400; Figure S4. FT-IR spectrum of _L_-Lysine ethyl ester diisocyanate (L-LDI); Figure S5. (a) GCMS of HMF, (b) HPLC of HMF; Figure S6. (a) GCMS of BHMF, (b) HPLC of BHMF; Figure S7. FT-IR spectra of the prepolymer and the final polyurethane PU; Figure S8. SEM image of the final polyurethane PU; Figure S9. Image of the final polyurethane PU tested for the removal of petroleum-derived fuels; Table S1. Langmuir dimensionless constant values.
