# Supplementary material for: Oil Spill Recovery of Petroleum-Derived Fuels Using a Bio-Based Flexible Polyurethane Foam
Source: Polymers (Basel). 2025 Jul 17;17(14):1959. doi: 10.3390/polym17141959 (PMC12298877; doi:10.3390/polym17141959)
Supplement: Supplementary file 1 [file polymers-17-01959-s001.zip › polymers-3727980-supplementary.pdf]

SUPPORTING INFORMATION

Oil Spill Recovery of Petroleum-Derived Fuels Using a Bio-Based Flexible Polyurethane Foam

Fabrizio Olivito [1,*], Zul Ilham [2,3*], Wan Abd Al Qadr Imad Wan-Mohtar [4], Goldie Oza [5], Antonio Procopio [6] and Monica Nardi [6]

[1] Department of Environmental Engineering, University of Calabria, Via P. Bucci, 87036 Arcavacata di Rende, Italy; fabrizio.olivito@unical.it (F.O.)

[2] Environmental Science and Management Program, Institute of Biological Sciences, Faculty of Science, Universiti Malaya, Kuala Lumpur 50603, Malaysia; ilham@um.edu.my (Z.I.Z.)

[3] Centre for Science and Environment Studies, Institute of Islamic Understanding Malaysia, 2 Langgak Tunku Off Jalan Tuanku Abdul Halim, 50480, Kuala Lumpur, Malaysia; ilham@um.edu.my (Z.I.Z.)

[4] Functional Omics and Bioprocess Development Laboratory, Institute of Biological Sciences, Faculty of Science, Universiti Malaya, 50603 Kuala Lumpur, Malaysia; qadyr@um.edu.my (W.A.A.Q.W-M)

[5] Centro de Investigation y Desarollo Tecnologico en Electroquimica Parque Tecnológico Querétaro, Queretaro, CP 76703, Mexico; goza@cideteq.mx (G.O.);

[6] Department of Health Sciences, University Magna Graecia of Catanzaro, Viale Europa - Campus Universitario S. Venuta - Loc. Germaneto, 88100 Catanzaro, Italy; procopio@unicz.it (A. P.); monica.nardi@unicz.it (M. N.);

Corresponding authors email address

fabrizio.olivito@unical.it; ilham@um.edu.my

Table of Contents

1.	**Regeneration tests in fuel/freshwater and seawater systems**	3
2.	**Freundlich isotherm plots**	6
3.	**Langmuir dimensionless constant**	9
4.	**Reagents**	10
5.	**Determination of the Isocyanate content of the prepolymer**	11
6.	**FT-IR spectrum of PEG 400**	12
7.	**FT-IR spectrum of L-Lysine ethyl ester diisocyanate (L-LDI)**	13
8.	**GC-MS and HPLC of HMF**	14
9.	**GC-MS and HPLC of BHMF**	15
10.	**FT-IR spetra**	16
11.	**SEM image of the final polyurethane PU**	17
12.	**Image of the final polyurethane PU**	18
13.	**References**	19

1) Regeneration tests in fuel/freshwater and seawater systems

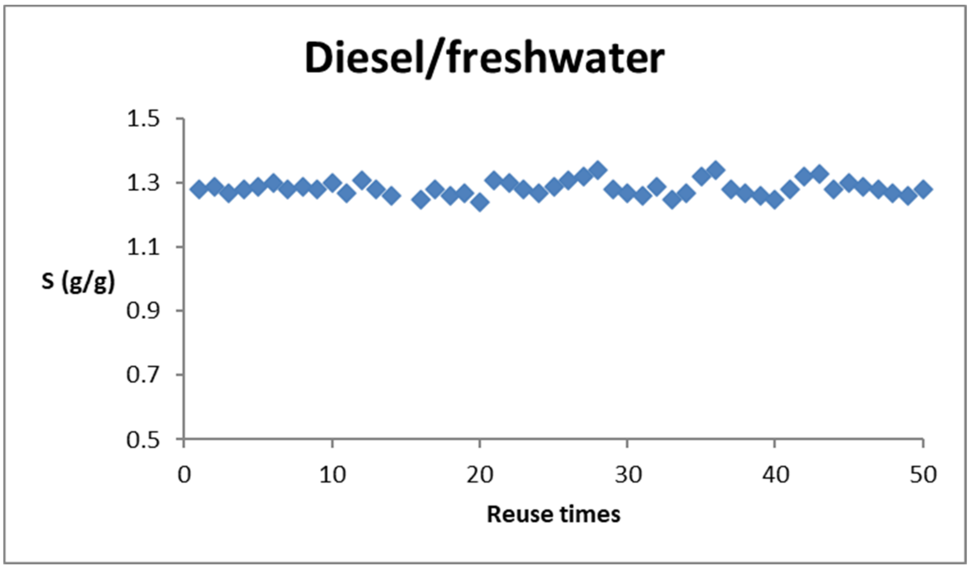

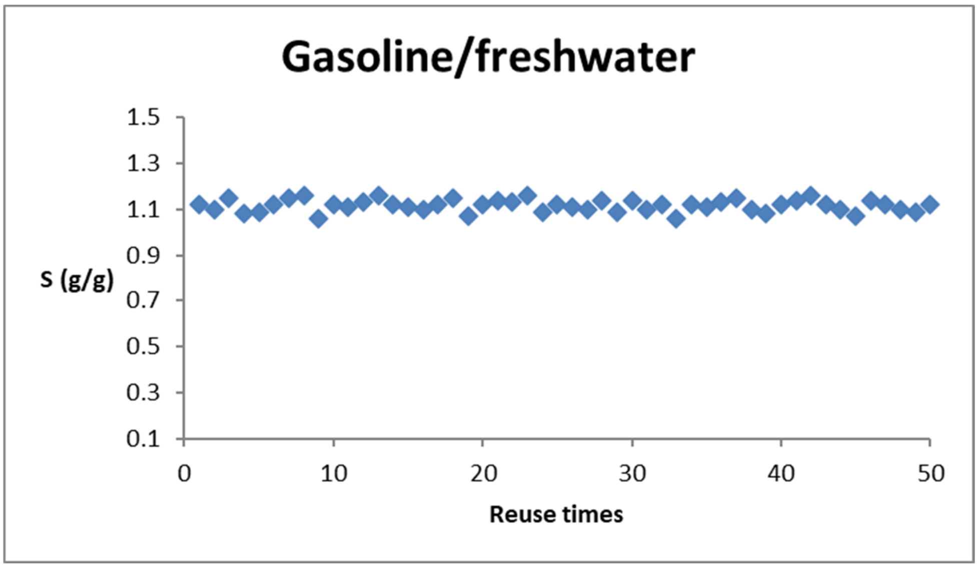

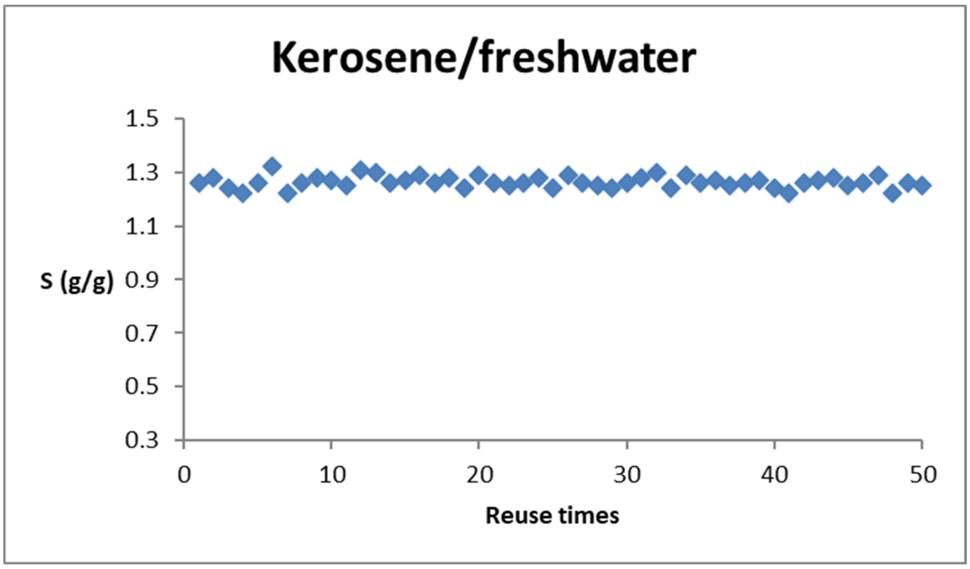

Kerosene/freshwater
1.5
1.3
1.1
S (g/g) 0.9
0.7
0.5
0.3
0
10
20
30
40
50
Reuse times

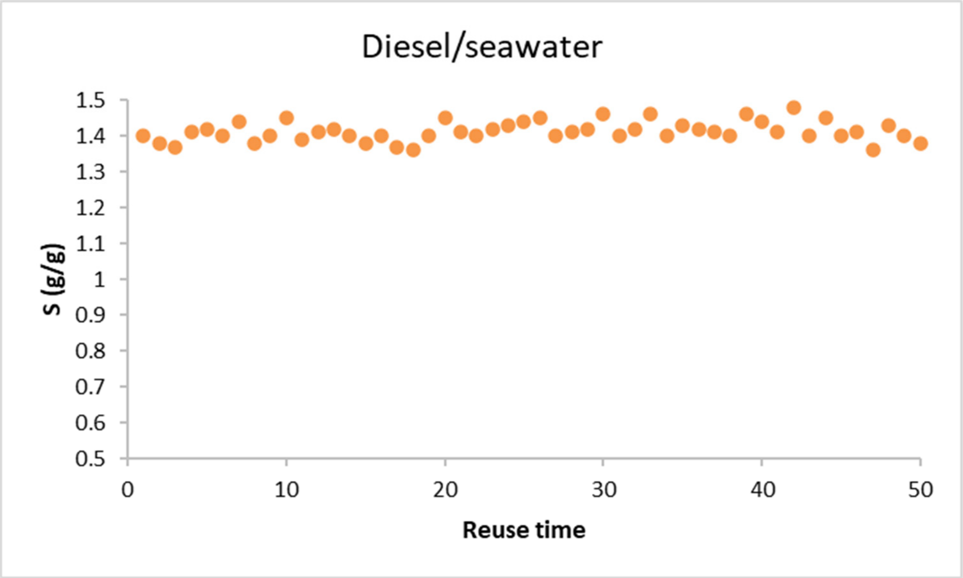

Diesel/seawater
1.5
1.4
1.3
1.2
1.1
1
0.9
0.8
0.7
0.6
0.5
S (g/g)
0
10
20
30
40
50
Reuse time

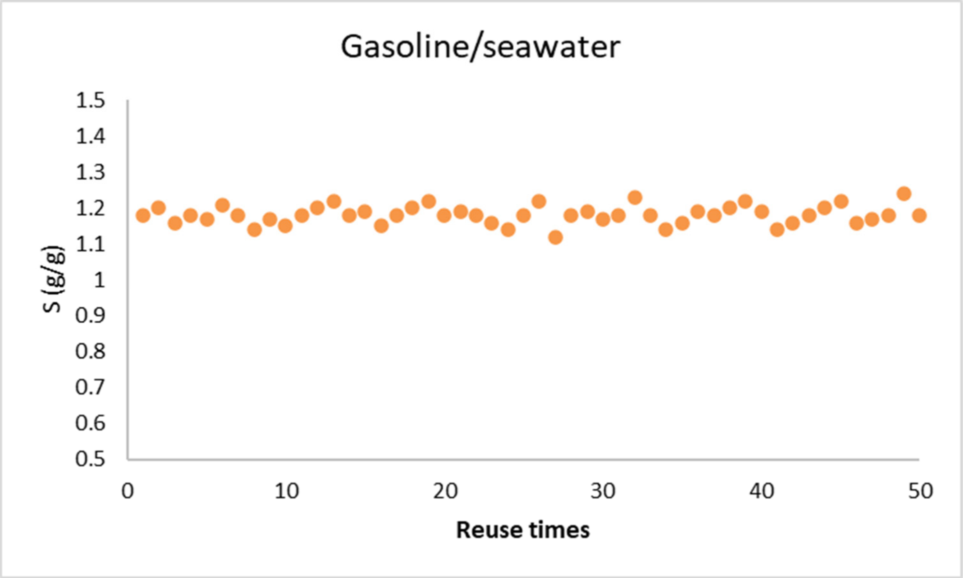

Gasoline/seawater
1.5
1.4
1.3
1.2
1.1
1
0.9
0.8
0.7
0.6
0.5
S (g/g)
0
10
20
30
40
50
Reuse times

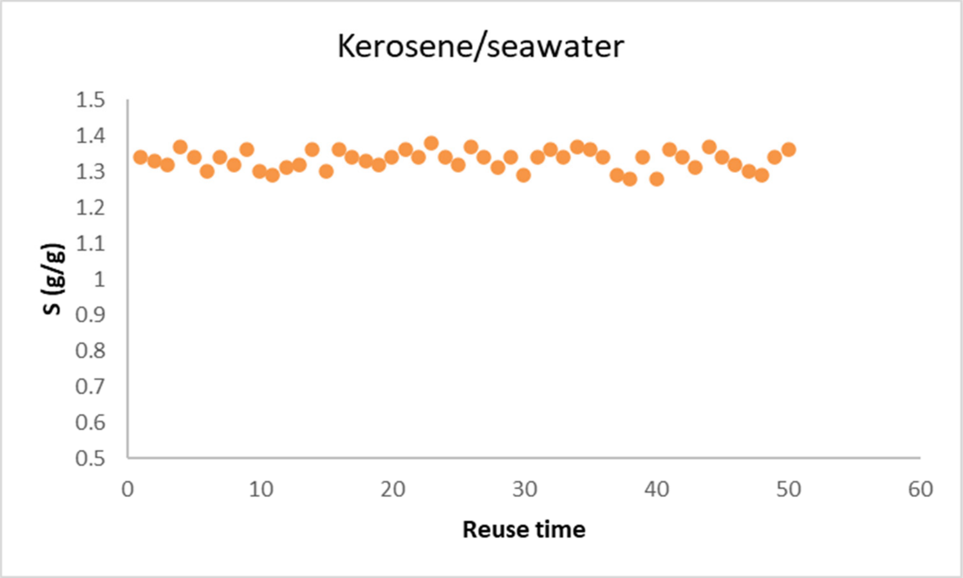

Fig. S1 Regeneration tests in fuel/freshwater and seawater systems

3) Freundlich isotherm plots

(a)

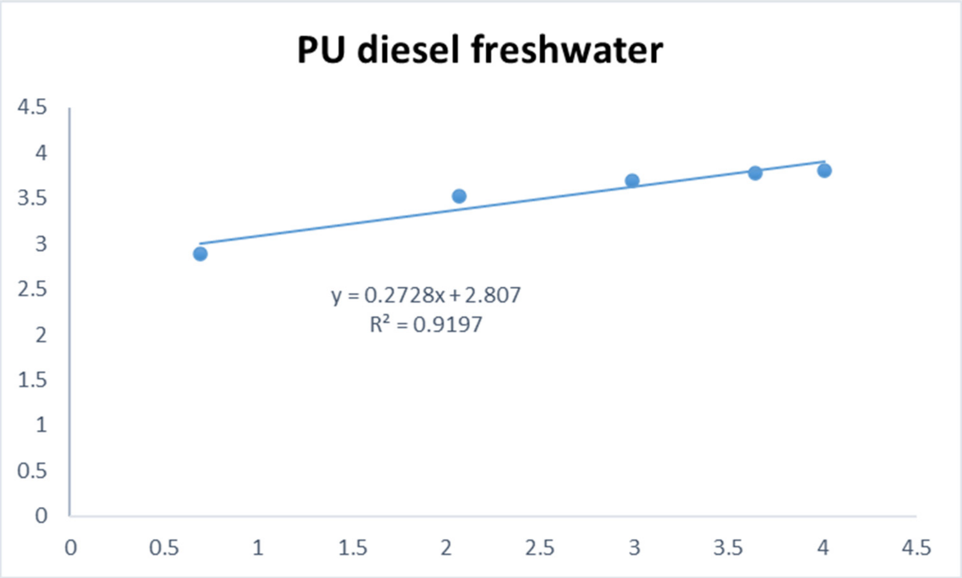

(b)

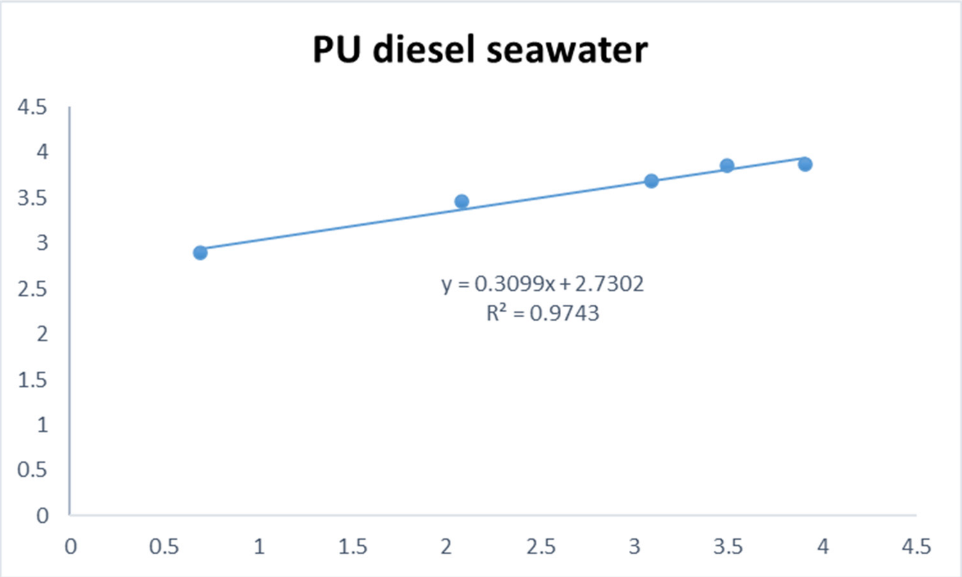

(c)

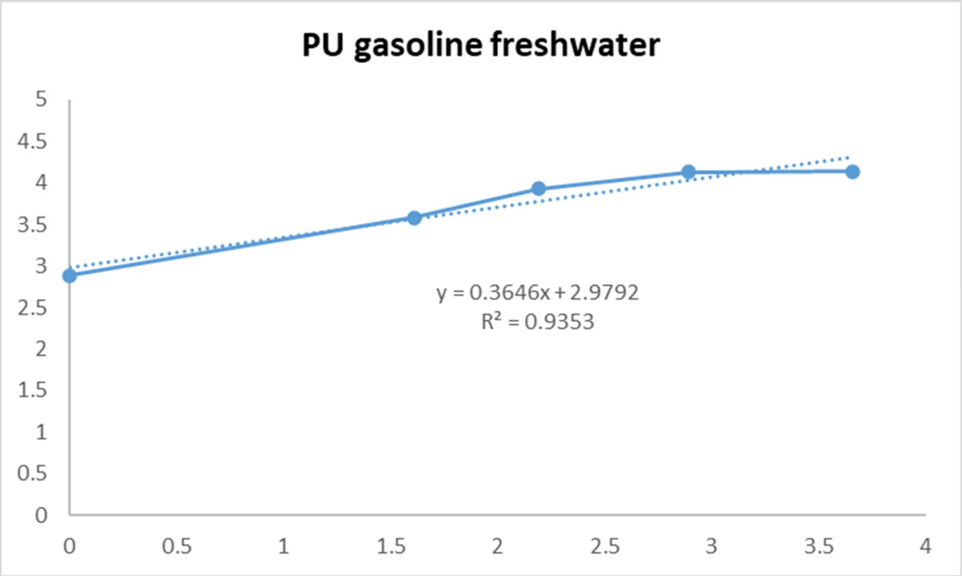
PU gasoline freshwater
5
4.5
4
3.5
3
2.5
2
1.5
1
0.5
0
0
0.5
1
1.5
2
2.5
3
3.5
4
y = 0.3646x + 2.9792
R² = 0.9353

(d)

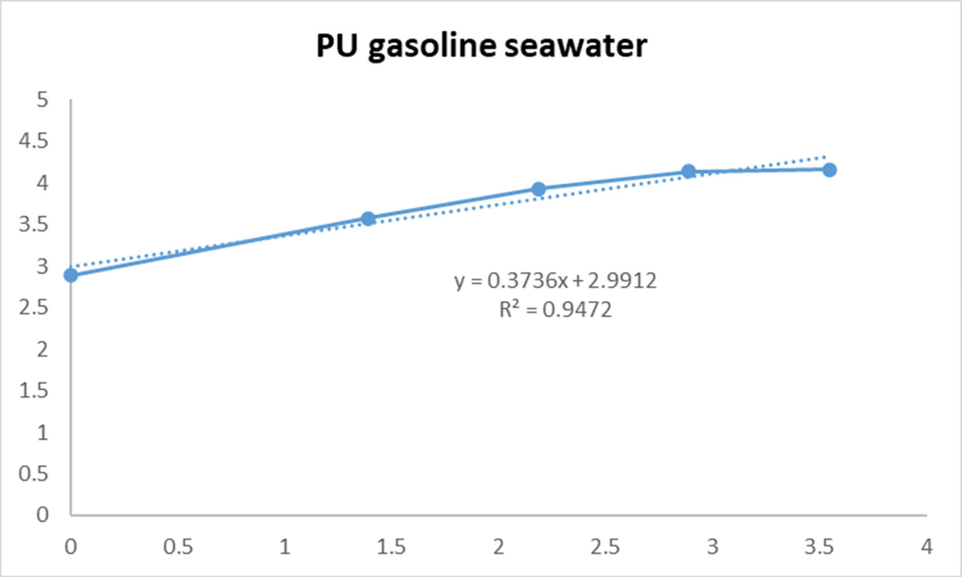
PU gasoline seawater
5
4.5
4
3.5
3
2.5
2
1.5
1
0.5
0
0
0.5
1
1.5
2
2.5
3
3.5
4
y = 0.3736x + 2.9912
R² = 0.9472

(e)

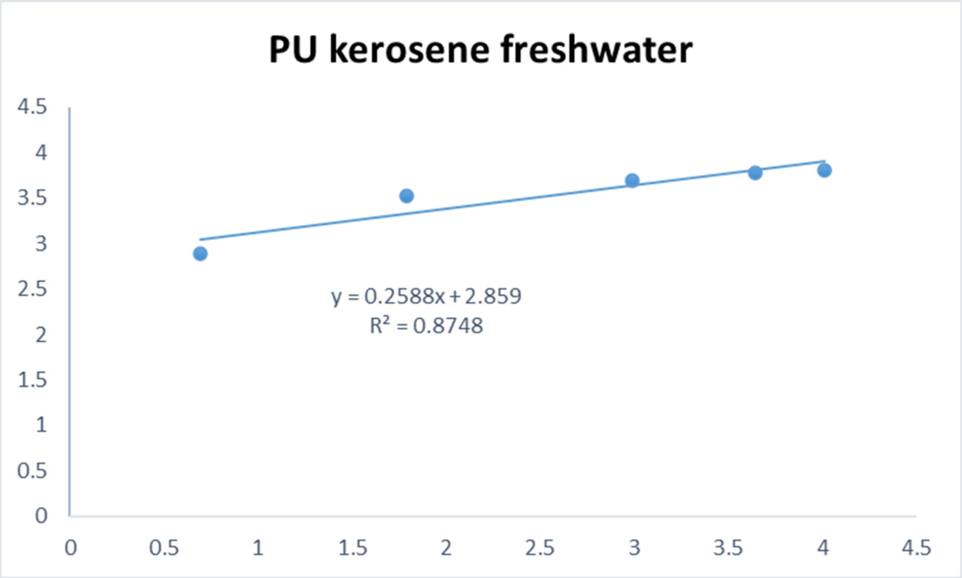

(f)

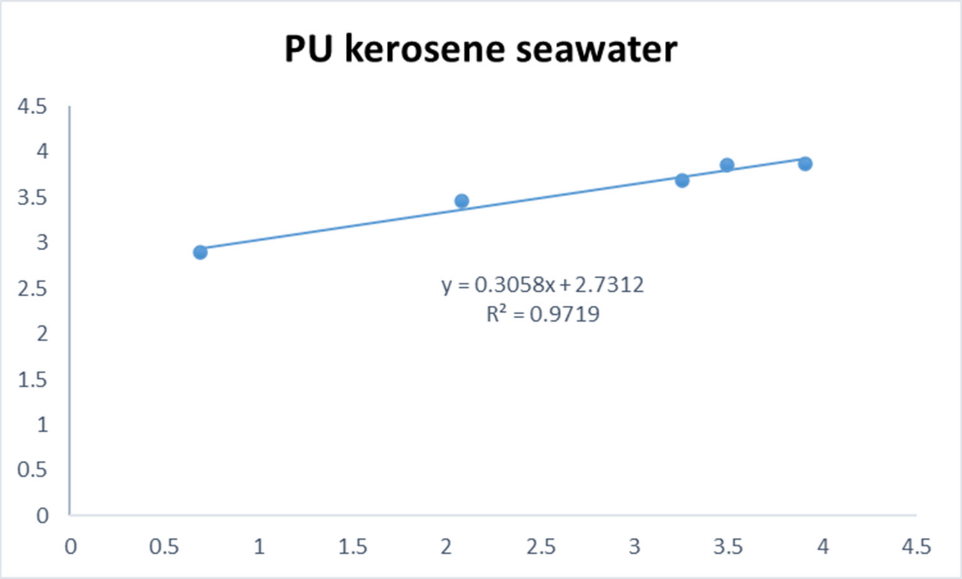

Fig. S2 (a) Freundlich plot for diesel/freshwater; (b) Freundlich plot for diesel/seawater; (c) Freundlich plot for gasoline/freshwater; (d) Freundlich plot for gasoline/seawater; (e) Freundlich plot for kerosene/freshwater; (f) Freundlich plot for kerosene/seawater

4) Langmuir dimensionless constant

One of the essential characteristics of the Langmuir isotherm can be expressed by a dimensionless constant, separation factor, R_L, defined as follows:

$$R_L = \frac{1}{1 + k_L C_0}$$

The value of R_L indicates the type of the isotherm; which is unfavourable ($R_L > 1$), linear ($R_L = 1$), favourable ($0 < R_L < 1$) or irreversible ($R_L = 0$).

In the following Table S1 we report the R_L values for each starting concentration of oil used:

C_0	Diesel/freshwater	Diesel/seawater	Gasoline/freshwater	Gasoline/seawater	Kerosene/freshwater	Kerosene/seawater
40	0.272	0.043	0.201	0.027	0.072	0.025
60	0.199	0.029	0.144	0.018	0.049	0.017
80	0.157	0.022	0.112	0.014	0.037	0.013
100	0.129	0.017	0.091	0.011	0.03	0.01
120	0.110	0.015	0.077	0.009	0.025	0.008

Table S1. Langmuir dimensionless constant values.

5. Reagents

Polyethylene glycol PEG 400
Molecular formula: $(C_2H_4O)n$
Appearance (Color): Clear colorless
Form: Viscous liquid
Identification (FTIR): Conforms
Molecular Weight: 62.07 (g/mol)

L-Lysine ethyl ester diisocyanate
Molecular formula: $C_{10}H_{14}N_2O_4$
Appearance (Color): Clear colorless
Form: liquid
Identification (FTIR): Conforms
Molecular Weight: 226.232 g/mol

6. Determination of the Isocyanate content of the prepolymer [i]

3-5 g of the prepolymer were dissolved in 50 ml of di-n-butylamine solution 0.2 N. 6 drops of bromophenol blue indicator were added. HCl 0.5 N was added until the colour of the solution turned to a yellow-green endpoint. The same procedure is carried out on the blank. The NCO % was calculated using the following formula:

$$\% \, NCO = \frac{4202\ (\mathrm{N})(\mathrm{B}-\mathrm{A})}{(sample\ weight)1000}$$

B = blank titer

A = sample titer

N = normality of the hydrochloric acid

*The prepolymer produced using the optimized reaction condition, present a NCO % of 15%, in accordance with the conventionally percentage reported industrially.[i]

7. FT-IR spectrum of PEG 400 [i]

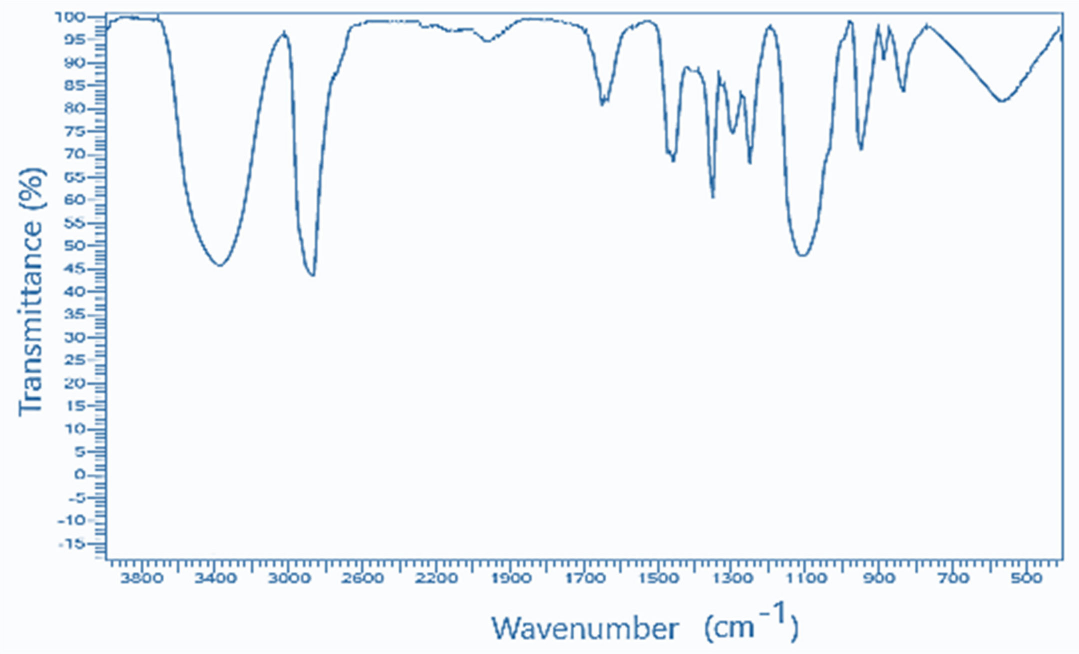

Fig. S3 FT-IR Spectrum of PEG 400

Functional group	Absorption peak (cm^{-1})
O-H	3379
Aliphatic C-H	2870
C-O-C and C-O-H	1103
Bending ethers	915-650

8. FT-IR spectrum of $_L$-Lysine ethyl ester diisocyanate (L-LDI) [i]

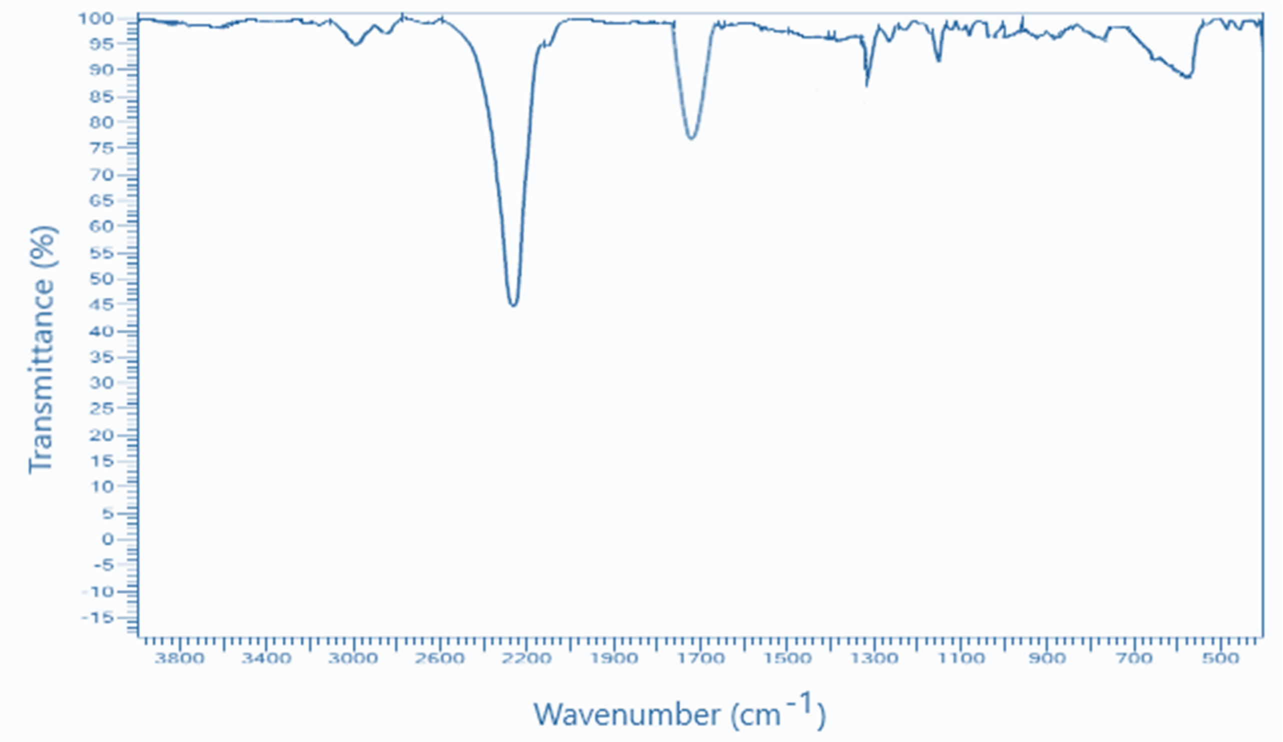

Fig. S4 FT-IR spectrum of $_L$-Lysine ethyl ester diisocyanate (L-LDI)

Functional group	Absorption peak (cm^{-1})
C-H stretching	2915
N=C=O stretching	2240
C=0	1740

9. GC-MS and HPLC of HMF [i]

GC-MS

(a)

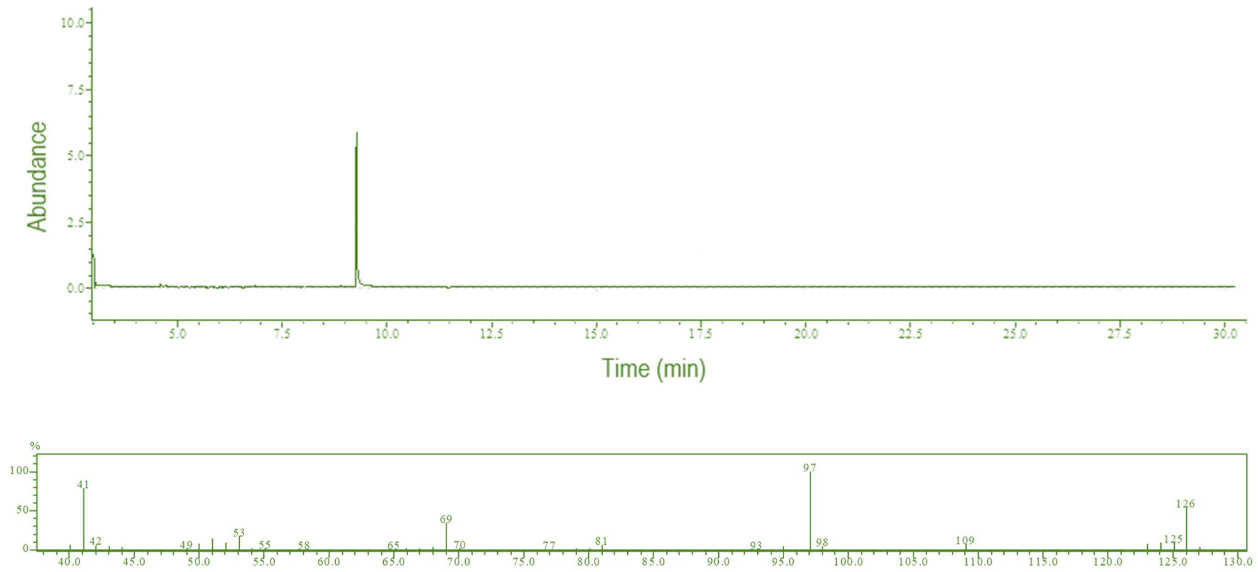

HPLC

(b)

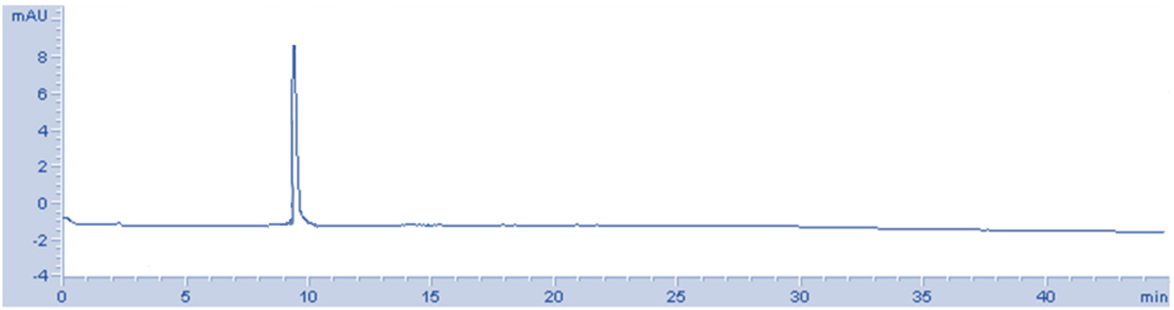

r.t. = 9.612

Fig. S5 (a) GCMS of HMF, (b) HPLC of HMF

10. GC-MS and HPLC of BHMF [i]

GC-MS

(a)

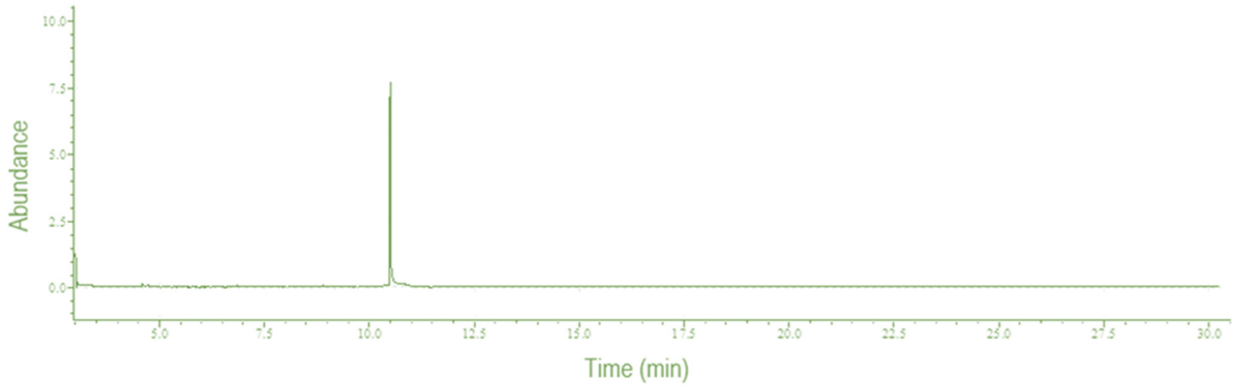

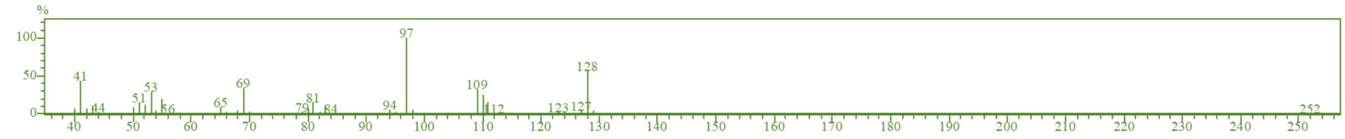

HPLC

(b)

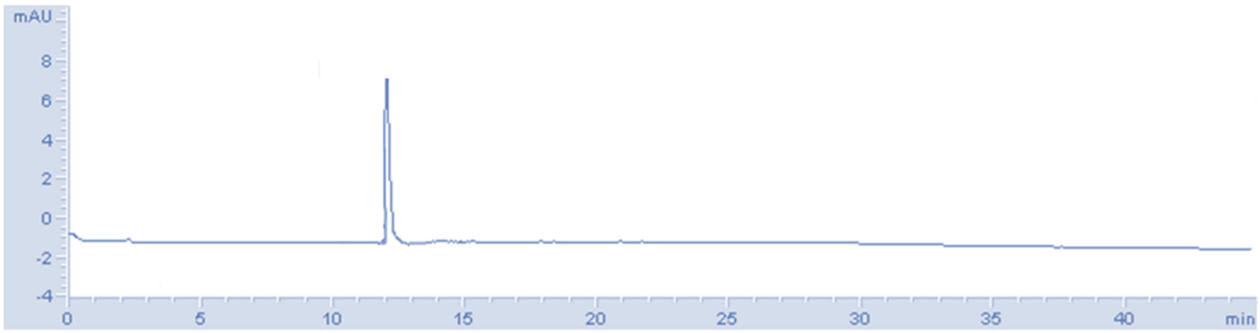

Fig. S6 (a) GCMS of BHMF, (b) HPLC of BHMF

11. FT-IR spectra [i]

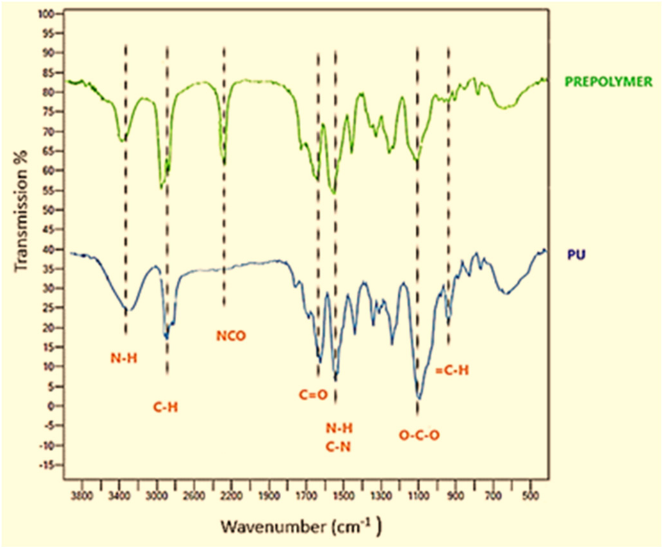

Fig. S7 FT-IR spectra of the prepolymer and the final polyurethane PU.

12. SEM [i]

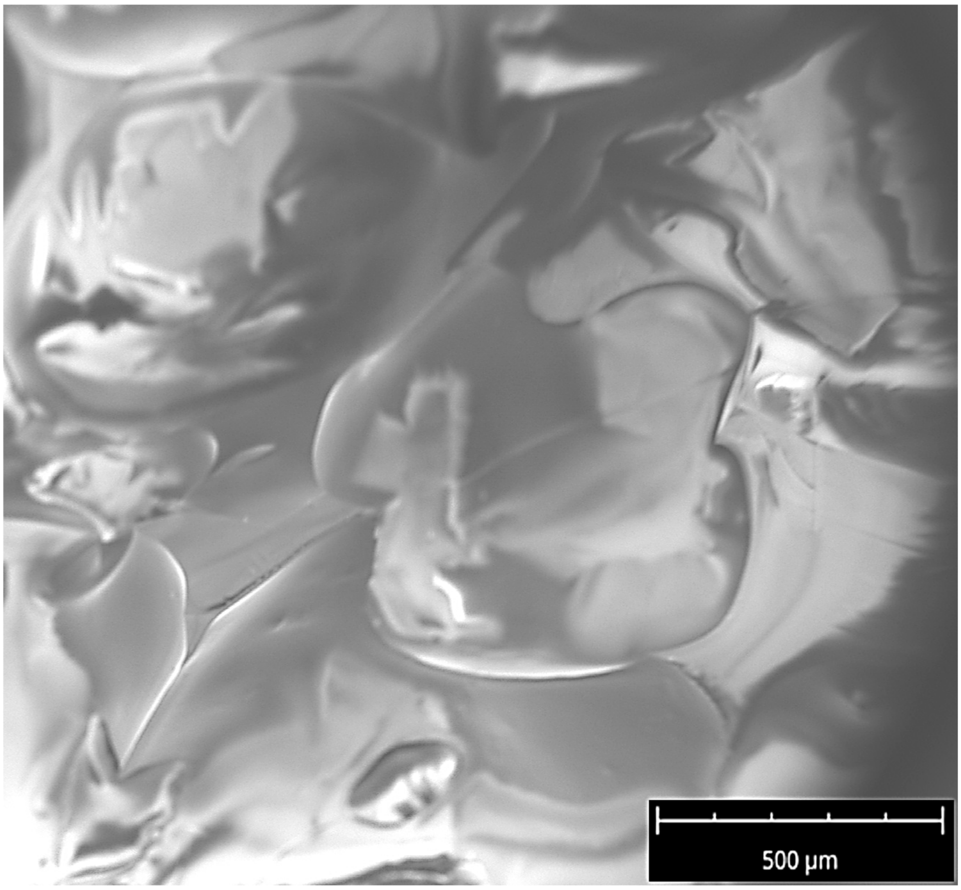

Fig. S8 SEM image of the final polyurethane PU

13. Image of the final polyurethane PU

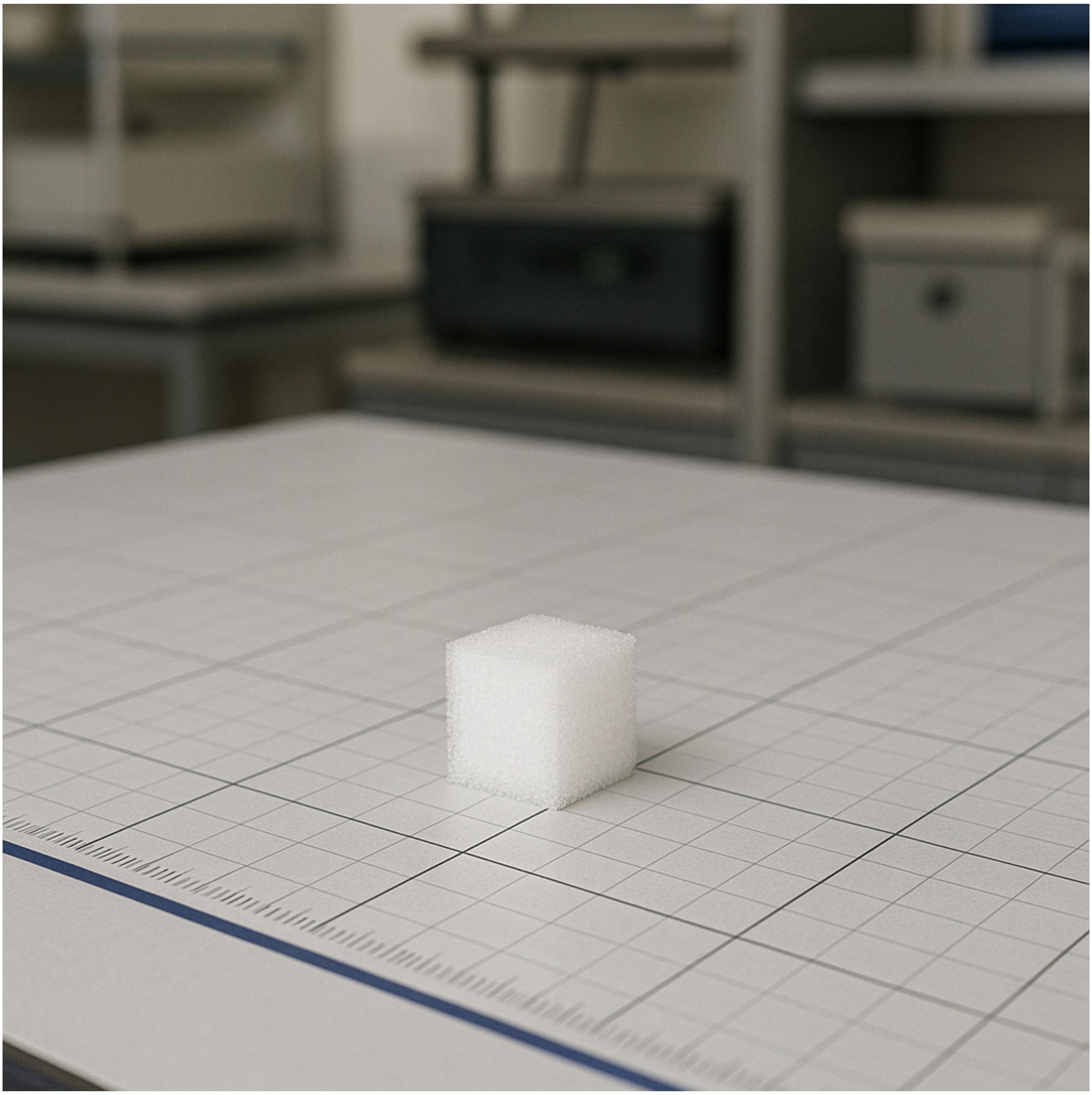

Fig. S9 Image of the final polyurethane PU tested for the removal of petroleum-derived fuels

14. References

[i] Olivito, F.; Jagdale, P.; Oza, G. Synthesis and Biodegradation Test of a New Polyether Polyurethane Foam Produced from PEG 400, L-Lysine Ethyl Ester Diisocyanate (L-LDI) and Bis-hydroxymethyl Furan (BHMF). Toxics 2023, 11, 698. https://doi.org/10.3390/toxics11080698
